# Supplementary material for: Computation of mixed resolvability for a circular ladder and its unbounded nature
Source: PLoS One. 2025 Mar 31;20(3):e0313788. doi: 10.1371/journal.pone.0313788 (PMC11957347; doi:10.1371/journal.pone.0313788)
Supplement: Attachment — Submitted filename: Response to Reviewer.docx [file pone.0313788.s001.docx]

**Response to Reviewers**

First and foremost, we want to convey our heartfelt gratitude to the reviewers for their insightful comments and ideas that helped us to improve the quality of this manuscript. We revised the manuscript according to the reviewer's report and editor's comments. In the following, we present replies to the reviewer suggestions one by one.

**Reviewer 1:**

Thank you very much Sir/Madam for pointing out all the mistakes in our manuscript.

*Revised correction (**blue line**).

1. Don’t use subject pronoun in abstract.

**Author Response:** We have now updated abstract of the manuscript.

2. Add more outcomes at the end of abstract.

**Author Response:** We have now added more data/findings in the abstract of the manuscript.

3. In introduction author said with size k and order m and used \left|E\right|\ =\ k\ and\ \left|V\right|\ =\ m. so don’t mention these terms two times.

**Author Response:** We have incorporated this, as suggested.

4. In introduction author should use number in ascending order for citation.

**Author Response:** We have updated the citation number in the ascending order as suggested.

5. Author should write table 2 instead of table no. 2.

**Author Response:** We have replaced “table no.” by “table” throughout the manuscript.

6. In reference list author should use same pattern for journal name.

**Author Response:** We have updated as well as corrected the reference list according to the journal style.

**Reviewer 2:**

Thank you very much Sir/Madam for pointing out all the mistakes in our manuscript.

*Revised correction (**blue line**).

1. I recommend changing the title of the research article as "Computation of mixed resolvability for a circular pentagonal ladder and its unbounded nature" making it more relevant and suitable in terms of conducted research.

**Author Response:** We have now updated the title of the manuscript as suggested.

2. The article lacks a clear motivation and fails to adequately explain the practical significance of studying this graph family. Although the study mentions applications in optimization, decision-making, and network design, it does not convincingly demonstrate how the theoretical findings translate to real-world problems. The discussion is largely confined to mathematical abstractions without connecting the results to concrete applications or providing examples of how the findings could be utilized in specific scenarios.

**Author Response:** First, the mixed metric basis and mixed metric dimension of the five-sided circular ladder, a planar graph, have not been studied previously. Therefore, we chose this graph to address this research gap, which serves as the primary motivation for our study. Further, adding to this we have explained properly the usefulness of studying resolvability parameters and planar graph in the introduction section (para no. 2 and last para of intro). Next, we appreciate the reviewer's observation regarding the lack of connection between the mathematical abstractions and concrete applications. While our current focus has been primarily on the theoretical aspects, we recognize the importance of demonstrating practical utility. We will consider exploring specific applications and providing real-world examples in future work to further highlight the practical significance of our findings.

3. I would like to suggest replacing some of the older cited references with more recent and relevant studies. Incorporating these up-to-date references will enhance the credibility and relevance of your research. Please consider integrating these recent studies as replacements for some of the older references in your manuscript.

**Author Response:** We have updated the literature by citing some of the above said studies in both introduction section as well as in the reference section of the manuscript.

4. The presentation of the theoretical results is somewhat redundant and repetitive in few places. The paper spends more time reiterating known concepts in metric dimension theory which can be omitted thereby directly referring them to those known results.

**Author Response:** We have tried to remove repetition of the known concepts in the metric dimension theory and have now referred to those results directly.
